# Genetic control of branching patterns in grass inflorescences

**DOI:** 10.1093/plcell/koac080

**Published:** 2022-03-08

**Authors:** Elizabeth A Kellogg

**Affiliations:** Donald Danforth Plant Science Center, St Louis, Missouri 63132, USA

## Abstract

Inflorescence branching in the grasses controls the number of florets and hence the number of seeds. Recent data on the underlying genetics come primarily from rice and maize, although new data are accumulating in other systems as well. This review focuses on a window in developmental time from the production of primary branches by the inflorescence meristem through to the production of glumes, which indicate the transition to producing a spikelet. Several major developmental regulatory modules appear to be conserved among most or all grasses. Placement and development of primary branches are controlled by conserved auxin regulatory genes. Subtending bracts are repressed by a network including TASSELSHEATH4, and axillary branch meristems are regulated largely by signaling centers that are adjacent to but not within the meristems themselves. Gradients of SQUAMOSA-PROMOTER BINDING-like and APETALA2-like proteins and their microRNA regulators extend along the inflorescence axis and the branches, governing the transition from production of branches to production of spikelets. The relative speed of this transition determines the extent of secondary and higher order branching. This inflorescence regulatory network is modified within individual species, particularly as regards formation of secondary branches. Differences between species are caused both by modifications of gene expression and regulators and by presence or absence of critical genes. The unified networks described here may provide tools for investigating orphan crops and grasses other than the well-studied maize and rice.

## Teaser

Recent work on grass inflorescence branching identifies extensive conserved regulation, but also divergence particularly in formation of secondary branches and spikelets.

## Introduction

Grass dominated ecosystems cover ∼40% of the Earth’s land surface (Lehmann et al., 2019; Griffith et al., 2020) and provide over 50% of the world’s calories, whether consumed directly or fed to animals which are then consumed by humans (FAO, 2003). Central to the ecological and economic roles of grasses is the inflorescence, the complex set of flowers that produces seeds. The combined number and size of seeds contribute to higher fitness in the wild and higher yield in cultivation. Accordingly, inflorescence structure and flower/seed production have been the target of both natural and human selection.

Grass inflorescence development is often described by invoking shifting meristem identity. As a shoot apical meristem (SAM) producing leaves on its flanks changes to producing bracts, branches, and floral meristems (FMs), it is described as acquiring inflorescence meristem (IM) identity. Within the inflorescence, similar shifts specify branch meristem (BM) identity and FM identity. In addition, in grasses a distinct structure, the spikelet (a tiny spike), is interpolated developmentally between the BM and FM and arises from a spikelet meristem (SM). The metaphor of meristem identity implies that the meristem is itself somehow autonomous and distinct from both the surrounding cells and also from other meristems on the plant.

In a thought-provoking paper, Whipple (2017) observed that the concept of meristem identity also implies the existence of selector genes whose presence confers particular characteristics on a meristem. While such selector genes are known for FMs, they have been elusive for other meristem types. Instead, he notes that the boundary between a meristem and its subtending bracts has emerged as an important signaling center (Whipple, 2017), shifting the metaphor from the meristem as a master controller of its own fate to the meristem as an emergent structure, the result of disparate inputs and outputs.

Another common metaphor is that of a developmental switch, a gene being either on or off. However, gene regulation is often quantitative, more like a rheostat than a binary switch, leading to metaphors of phase change (e.g. Kyozuka, 2014) and gradual transitions from one state to another. At the beginning and end of the transition, a structure may be recognizable as an IM, BM, or SM, but the boundaries between them may not be sharp, although a gradual transition might ultimately trigger a switch. These metaphors—identity and signaling centers, switches, and rheostats—currently co-exist productively and are themes that occur throughout this review.

This review focuses on a narrow but critical developmental window, from the production of primary BMs by the IM through to specification of spikelets. These are processes that vary extensively in response to natural (evolutionary) and human (agricultural) selection. Many of the genes mentioned here have additional functions in vegetative growth and in spikelet development, but pleiotropy may obscure their role in any single process, hence the focus on a narrow slice of time. Specifically, I do not address the vegetative–reproductive transition and control of flowering time, which are thoroughly discussed elsewhere (e.g. Distelfeld et al., 2009; Lee and An, 2015; Doust et al., 2017; Woods et al., 2019), nor do I review the fundamental controls of IM size, which are also covered in recent reviews (e.g. Bommert and Whipple, 2018). The extensive data on floret structure, form, and function (e.g. Schrager-Lavelle et al., 2017; Shen et al., 2021) are also not covered here. Some of these topics are included in the related review by Richardson and Hake (2022), which focuses particularly on the incomparable data available from maize, and also recent species-focused reviews on rice (Li et al., 2021a, 2021b, 2021c) and Triticeae (Gao et al., 2019; Gauley and Boden, 2019; Sakuma and Schnurbusch, 2020). Protein-coding genes discussed in this review are listed in Supplemental Table S1.

## Grass inflorescences are branched structures with branches producing spikelets

**Figure 1:**
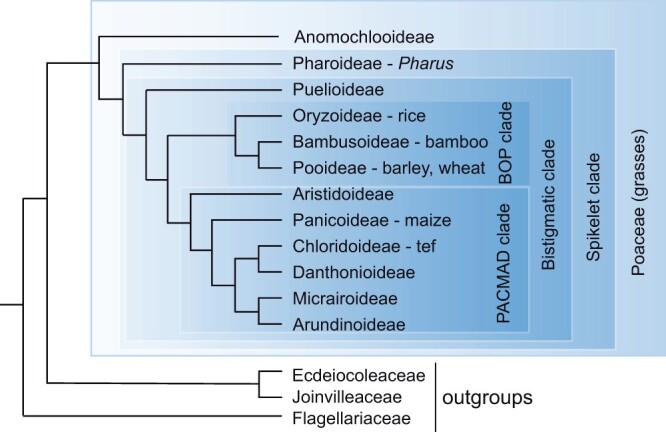
Phylogeny of the grass family summarized from GPWG II (2012); Soreng et al. (2017), and Saarela et al. (2018). Within the grasses, terminal taxa are subfamilies; representative crops are given where applicable.

The grass family (Poaceae or Gramineae, both correct names) includes about 700 genera and 12,000 species (Kellogg, 2015; Soreng et al., 2017). The family is divided into 12 subfamilies, 3 of which (Anomochlooideae, Pharoideae, and Puelioideae) are successive sisters to the remainder of the family and together include only a handful of species (GPWG II, 2012; Saarela et al., 2018; Figure 1). The other nine subfamilies fall into two large clades that are strongly supported by phylogenetic data and are named by the acronym for the included subfamilies (Kellogg, 2015; Soreng et al., 2017). The BOP clade (with ∼40% of the species in the family) includes Bambusoideae, Oryzoideae, and Pooideae, while the PACMAD clade (with ∼60% of the species) includes Panicoideae, Aristidoideae, Chloridoideae, Micrairoideae, Arundinoideae, and Danthonioideae.

**Figure 2:**
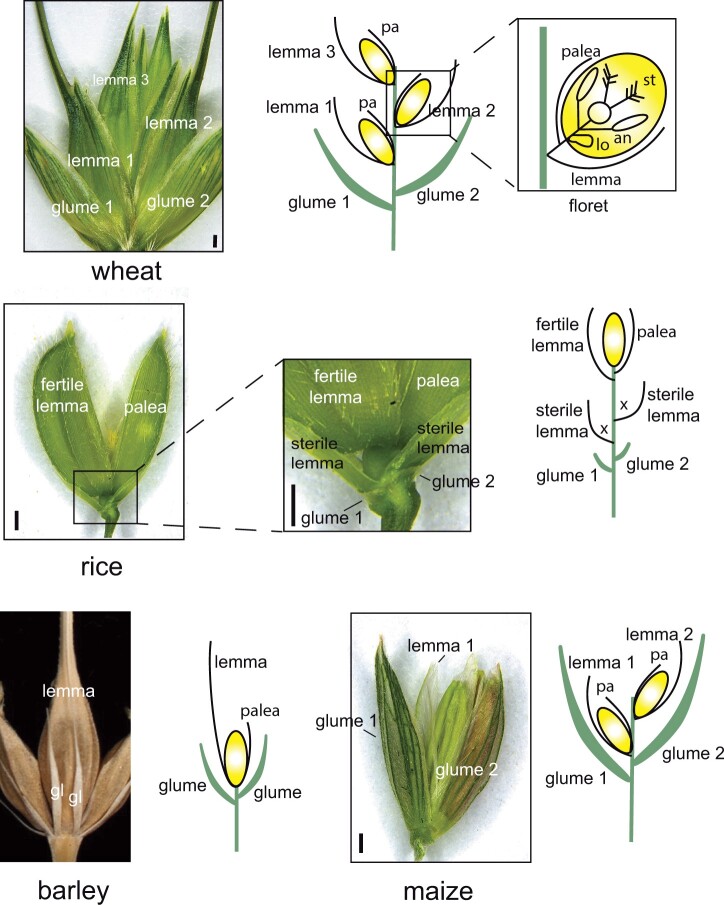
Images and diagrams of spikelets of wheat (*Triticum turgidum* cv. “Kronos”), rice (*O. sativa*), barley (*H. vulgare*, only central spikelet diagrammed), and maize (*Z. mays*, tassel spikelet). Paleas not visible in photos of wheat and barley. Shaded ovals (yellow) indicate floral organs as shown in inset, upper right. Glumes indicated by thickened arcs (green); lemmas and paleas, black arcs; suppressed meristems, x. Scale bars, 0.5 mm. Distance between structures in diagrams is exaggerated for clarity. Image of barley spikelet reproduced from Komatsuda et al. (2007); copyright 2007 National Academy of Sciences. Scale bar lacking in original. an, anther; gl, glume; lo, lodicle; pa, palea; st, stigma.

Grass inflorescences are distinctive. In all but the four species of Anomochlooideae (Judziewicz and Soderstrom, 1989), the flowers (florets) are borne in units known as spikelets (GPWG, 2001; Figure 2). Each spikelet consists of sterile bracts (glumes, generally two) and a spikelet axis bearing one or more florets. The number of florets per spikelet is generally fixed within a species or clade or varies within a narrow range. The flowers themselves are zygomorphic, with a large subtending bract (the lemma), in the axil of which is a conventional, if highly reduced, flower with an outer perianth (the palea), inner perianth (lodicules), stamens, and a gynoecium with a plumose stigma and single ovule. Florets are thus determinate structures and their formation marks an end to any further branching processes.

Grass inflorescences are often described as spikes, racemes, or panicles, borrowing terminology from dicots and nongrass monocots. However, because the terminal units are spikelets, which are themselves inflorescences, rather than flowers as in dicots, the grass inflorescence is in fact a compound structure, an inflorescence of spikes, and is therefore technically a synflorescence (Weberling, 1989; Vegetti and Weberling, 1996). I will use the more common term inflorescence here even though it is somewhat inaccurate.

**Figure 3:**
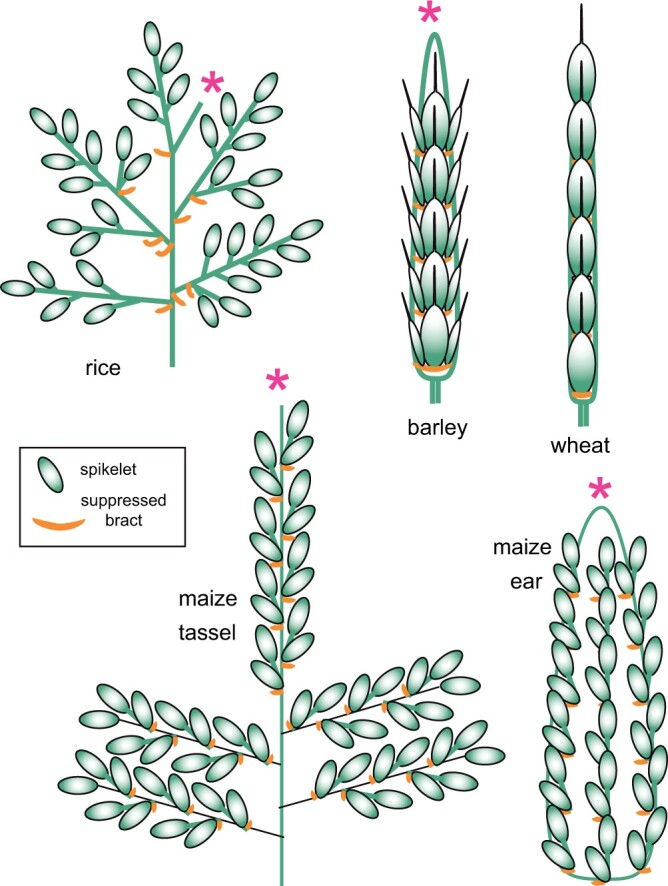
Inflorescence diagrams of rice (*O. sativa*), barley (*H. vulgare*), wheat (*T. aestivum*), and maize (*Z. mays*). Spikelets indicated by ovals (green); suppressed bracts, arcs below the ovals (orange). IMs lacking a terminal flower indicated by asterisks (magenta).

In this review, I treat the grass family as a single genetic system (Bennetzen and Freeling, 1993), with insights coming from cross-species comparisons as much as from detailed studies in a single species. Data come largely from rice (*Oryza sativa*, tribe Oryzeae, subfamily Oryzoideae) and maize (*Zea mays*, tribe Andropogoneae, subfamily Panicoideae), barley, and wheat (*Hordeum vulgare* and *Triticum aestivum*, respectively, tribe Triticeae, subfamily Pooideae; Figures 1–3; gene names in Supplemental Table S1), although I will also mention *Brachypodium distachyon* (tribe Brachypodieae, subfamily Pooideae), and green millet (*Setaria viridis*, tribe Paniceae, subfamily Panicoideae). No universal system of gene nomenclature exists for the grasses, so for consistency gene and protein names are written in all capital letters, with the gene names italicized. I do not try to distinguish orthographically between dominant and recessive alleles.

## Inflorescence architecture is the outcome of a repeating series of developmental decisions

The pattern of inflorescence architecture is governed by the relative timing of shifts from branch-producing to spikelet-producing meristems, what Kyozuka (2014) has described as a “meristem phase change.” In all grasses after the transition of the SAM to reproductive activity, an IM or BM has only three possible fates: (1) it can function as a branch-producing meristem, with new BMs arising on its flanks; (2) it can form a spikelet (SM), or (3) it can cease to function, with cell division and growth coming to a halt. If the BM produces higher order BMs, these in turn have the same set of developmental options. Production of glumes marks the transition from a BM to an SM, and further development is canalized to produce one or more florets.

The inflorescence architecture of grasses is well documented and shows that the number of iterations of these developmental decisions varies among species and genera. However, for any given species, the number of BMs produced by an IM or other BMs is fixed within a narrow range as is the number of SMs.

Inflorescence development may be modeled as a series of on–off switches, with shifting patterns of identity (Kellogg, 2000). In contrast, Prusinkiewicz et al. (2007) and Harder and Prusinkiewicz (2013) describe a model more similar to a rheostat with continually varying amounts of what they term “vegetativeness.” The models are not mutually exclusive, in that the switch model can be viewed as a simple version of the rheostat model. However, the latter model has never been elaborated formally to accommodate the complexities of grass inflorescence architecture.

The branching pattern of grass inflorescences is established early in development when the IM is still enclosed by the sheathing leaf bases. Elongation of inflorescence internodes occurs later (Patil et al., 2019; McKim, 2020; E. A. Kellogg, personal observation). Most research has focused on branching patterns because they determine the number of florets and hence the number of grains, and this review reflects that same bias, with few comments on elongation. Nonetheless, inflorescence elongation patterns may have fitness consequences in both natural and agricultural settings, for example, by determining whether the florets must pollinate themselves or can shed pollen away from the plant, or determining the propensity of the inflorescence stalk to break or be attacked by herbivores.

Whether the IM ultimately produces a spikelet is independent of whether the primary BMs terminate in spikelets or simply cease growing. The IM ceases producing BMs and ends as an undifferentiated dome or axis in maize (*Z.* *mays*), rice (*O.* *sativa*), signal grass (*Brachiaria decumbens*), and finger millet (*Eleusine coracana*; Figure 3), whereas it ultimately becomes an SM in wheat (*T.* *aestivum*), ryegrass (*Lolium* spp.), sorghum (*Sorghum bicolor*), and oats (*Avena sativa*; Butzin, 1979; Moncur, 1981; Liu et al., 2007; Reinheimer and Vegetti, 2008; Reinheimer et al., 2009, 2013; Kellogg et al., 2013). While presence of a terminal spikelet is consistent within a species or genus, it is labile in evolutionary time (Reinheimer and Vegetti, 2008; Reinheimer et al., 2013; Kellogg, 2015), suggesting it can be altered easily by natural selection but is generally not a plastic response to the environment.

## Placement of bracts is governed by auxin and specifies branching patterns

### The role of auxin

Branches in all plants form from meristems in the axils of leaves or bracts, which in turn are controlled by local auxin maxima leading to lateral organ production (Reinhardt et al., 2003; Smith et al., 2006). In rice and maize, auxin accumulates in all BMs of the inflorescence, as well as in the primordia of glumes and other floral organs (Yang et al., 2017). Auxin biosynthesis, transport, and signal transduction have been recently reviewed by Matthes et al. (2019), who provide detailed information on the molecular evolution and expression of auxin-related genes in rice and maize and compare auxin biology in grasses with that in Arabidopsis. Bract and axillary branch formation require auxin biosynthesis, as shown by disruption of the auxin biosynthetic genes in maize *VANISHING TASSEL2* and *SPARSE INFLORESCENCE1* and their rice orthologs *OsTAR2/OsFISHBONE* and *OsYUC1* (Gallavotti et al., 2008; Phillips et al., 2011; Yoshikawa et al., 2014; Matthes et al., 2019). All are expressed in axillary meristems of the inflorescence.

**Figure 4:**
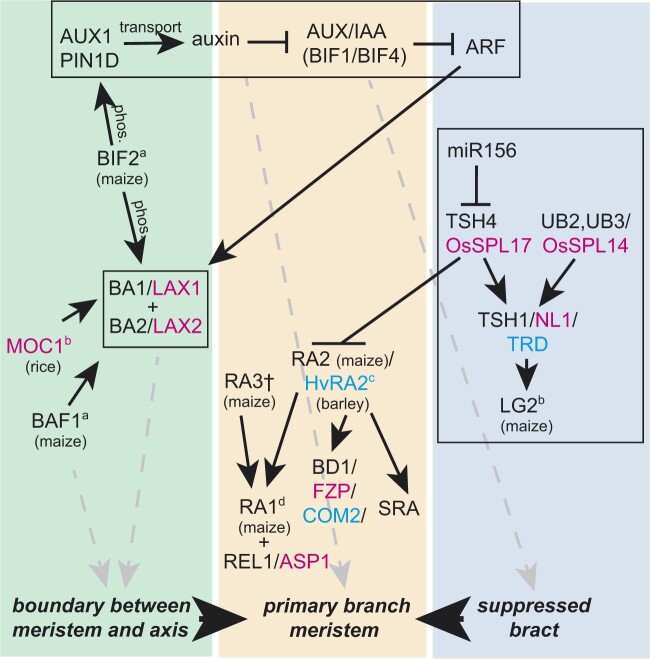
BA1/LAX1 (boundary domain), RA2 (axillary meristem), and TSH4 (suppressed bract) regulatory networks. Subnetworks in boxes are widely conserved among the grasses. ^a^Ortholog in rice not involved in regulating LAX1LAX2. ^b^No data on orthologs in other grasses. ^c^RA2/HvRA2 are expressed adjacent to the meristem but not overlapping with BA1 + BA2. ^d^Gene absent in rice and barley genomes. Dashed lines indicate regulatory connection inferred from gene expression and mutant phenotypes, following Yao et al. (2019). For genes with different names in different species, the first name is the maize gene name (black), second is rice (magenta), and third is barley (cyan).

Auxin flux through the epidermis converges on small regions of the IM, a process regulated by the auxin influx carrier ZmAUX1 (SvAUX1 in *S.* *viridis* (Zhu et al., 2021a, 2021b) and the auxin efflux carrier SISTER OF PINFORMED1 (SoPIN1/ZmPIN1D; O'Connor et al., 2014; Matthes et al., 2019; Figure 4). Loss-of-function mutations in *SvAUX1*/*SPARSE PANICLE1* reduce all orders of branching in the inflorescence, whether primary, secondary, tertiary, or higher, although the effect in maize is less striking than in *S. viridis* (Huang et al., 2017; Zhu et al., 2021a, 2021b). Vein patterning is controlled by PIN-FORMED1a (PIN1a) and PIN1b, which move auxin away from the local maxima and establish the position of vascular tissue (O'Connor et al., 2014). SoPIN1/ZmPIN1D is shared by all angiosperms except Brassicaceae, where the gene appears to have been lost (O'Connor et al., 2014; Matthes et al., 2019). In rice, mutations in *OsPIN1a* and *OsPIN1b* primarily affect the root system, although *PIN1a* mutants and the double mutant *PIN1a PIN1b* exhibit increased branch angle in the inflorescence (Li et al., 2019a, 2019b). The single mutants *PIN1c* and *PIN1d* had no effect on the inflorescence, but the double *PIN1c PIN1d* mutant abolished all inflorescence branching (Li et al., 2019a, 2019b). The wheat TaPIN1proteins also affect spikelet number and grain number per inflorescence (Yao et al., 2021).

ZmPIN1a is phosphorylated by BARREN INFLORESCENCE2 (BIF2), a homolog of Arabidopsis PINOID (McSteen et al., 2007; Skirpan et al., 2008). Without BIF2, branches do not initiate. In rice, mutations of OsPID had no effect on inflorescence branching although they affected floral organ development (Xu et al., 2019).

The auxin/indole-acetic acid (Aux/IAA) proteins form a large complex family of proteins in the grasses (as in other plants), but appropriate genetic studies are largely lacking. Few AUX/IAA mutants are known to have clear effects on inflorescence branching (Matthes et al., 2019). Two such genes in maize, *BIF1*/*ZmIAA27* and *BIF4*/*ZmIAA20*, are expressed in the IM and in the central zone of axillary meristems (Galli et al., 2015). BIF1 and BIF4 interact with maize activating auxin response factors (ARFs) and appear to regulate the placement of axillary meristems and to repress their formation elsewhere.

### Phyllotaxis, a read-out of hormonal signals

In most grasses and their outgroups, the IM produces bracts and their axillary primary branches in a spiral, a shift from the distichous phyllotaxis of the vegetative shoot (Kellogg et al., 2013; Bartlett and Thompson, 2014). Although spiral inflorescence phyllotaxis is apparently ancestral in the grasses, all members of Pooideae except the early diverging genus *Brachyelytrum* (Kellogg et al., 2013), some Danthonioideae and many Andropogoneae (Panicoideae) maintain two-ranked bracts and branches in the inflorescence. Also in Pooideae, some inflorescences are two-ranked but with a divergence angle ˂180°, a pattern that is phenotypically similar to rice inflorescences with mutations in *ABERRANT PANICLE ORGANIZATION1* (*APO1*; Ikeda et al., 2005, 2007). In such inflorescences, the placement of the bracts does not follow the placement of the leaves (Kellogg et al., 2013). Truly distichous inflorescences appear in the crown Pooideae (tribes Brachypodieae, Triticeae, Bromeae, and Poeae).

## Inflorescence bracts are suppressed; axillary meristem growth is continuous

### Bract suppression

In nearly all grasses, inflorescence bracts are suppressed and are apparent only in early development as narrow ridges (see e.g. Moncur, 1981; Kellogg et al., 2013), a pattern distinct from most other angiosperms and grass outgroups in which inflorescence bracts expand. Conversely, axillary branches in grass inflorescences grow immediately upon initiation (Kyozuka 2014; Li et al., 2019a, 2019b). Inflorescence bract suppression contrasts with that of vegetative growth, in which the leaves expand but the axillary meristems generally fail to grow out immediately (Kyozuka 2014). However, bracts do expand in some parts of the inflorescence of Bambusoideae, many Andropogoneae, and possibly in *Anomochloa*, although the morphology of the latter is complex and hard to interpret (Judziewicz and Soderstrom, 1989; Judziewicz et al., 1999; Sajo et al., 2012).

The proteins TASSELSHEATH1 (TSH1) and TSH4 repress inflorescence bracts; when both are mutated, bracts expand and axillary meristems are reduced or entirely absent (Whipple et al., 2010; Xiao et al., 2021). TSH1 is a GATA-domain zinc finger transcription factor orthologous to NECKLEAF1 (NL1) in rice and THIRD OUTER GLUME (TRD) of barley (Houston et al., 2012), which have similar mutant phenotypes and together are the founding members of the NL1/TSH1/TRD (NTT) family of proteins. TSH4 is a transcription factor in the SQUAMOSA-PROMOTER BINDING (SBP)-LIKE (SPL) family; it is orthologous to ZmSBP23 and the two maize proteins are co-orthologous to OsSP17 (Wei et al., 2018).

Both *TSH4* and *TSH1* are expressed in the cryptic bract of inflorescence branches and act synergistically, with *TSH4* upstream of *TSH1* (Xiao et al., 2021; Figure 4). Together they regulate the expression of *LIGULELESS2* (*LG2*), encoding a basic leucine zipper protein that regulates outgrowth of long branches among many other phenotypes (Walsh and Freeling, 2002). *TSH1* is co-expressed with *ZmYABBY15*, a gene expected to be expressed in all leaf-like organs, confirming that the minute structure is indeed a bract. Based on gene expression and mutant phenotypes, Xiao et al. (2021) suggest that the maize SPL proteins UNBRANCHED2 (UB2) and UB3 may also help regulate *TSH1* expression. UB2 and UB3 are co-orthologous to OsSPL14 (Chuck et al., 2014) and may control allocation of cells to lateral organs in general.

TSH1 and the NTT proteins NTT-like1 and NTT-like2 all share a HAN domain with the dicot proteins related to HANABA TARANU (HAN; Whipple et al., 2010), although the NTT proteins are all more closely related to each other than any of them is to the dicot proteins. Despite the sequence similarities among grass (NTT) and dicot HAN proteins, HAN does not regulate bract development, even though bracts are suppressed in Brassicaceae. Thus, bract suppression in the grasses must proceed by a mechanism quite different from that of Arabidopsis (Whipple et al., 2010). NTT-like genes are expressed in boundary regions in outgroups of the grasses, but early in grass evolution *TSH1* acquired SBP binding sites in its promoter, bringing it under the control of TSH4 and causing bract expression (Xiao et al., 2021). In this position, TSH1 retains its presumed ancestral developmental role of suppressing cell division and growth, as well as a possible role in signaling.

### Axillary meristem growth: the BA1/LAX1 network

Signaling centers adjacent to axillary meristems appear to specify which cells develop as part of the bract, which become part of the meristem, and which cease dividing entirely and form a boundary. Regulatory proteins in these centers likely also activate mobile factors that then move into the meristem (Whipple, 2017). Such regulatory proteins include a conserved basic helix–loop–helix transcription factor, LAX PANICLE1 (LAX1 in rice, orthologous to BARREN STALK1, BA1, in maize) that is required for axillary branch formation (Komatsu et al., 2003a, 2003b; Gallavotti et al., 2004; Figure 4). In all grasses that have been investigated, the underlying genes are expressed in the boundary between the axillary meristem and the axis that bears it (Komatsu et al., 2003a, 2003b; Gallavotti et al., 2004; Oikawa and Kyozuka, 2009; Woods et al., 2011). BA1/LAX1 mutations do not affect bract formation so are specific to the region just adaxial to the meristem. The auxin transport protein BIF2 (OsBIF2 in rice) physically interacts with and phosphorylates BA1 (Skirpan et al., 2008).

BA1/LAX1 interacts directly with BA2/LAX2, a nuclear-localized protein that is conserved in grasses (Figure 4). The expression domains and mutant phenotypes of *BA1/LAX1* and *BA2/LAX2* overlap, further supporting their involvement in the same pathway (Tabuchi et al., 2011; Yao et al., 2019). *BA1/LAX1* is expressed in *BA2/LAX2* mutants and vice versa, indicating that the proteins do not regulate each other’s transcription (Tabuchi et al., 2011; Yao et al., 2019).

Other regulators of *BA1/LAX1* have been identified in maize and rice but have been investigated in only one of the two species. For example, MONOCULM1 (MOC1) in rice, a transcription factor with a GRAS domain similar to that of LATERAL SUPPRESSOR in Arabidopsis, positively regulates *LAX1* (Li et al., 2003). *MOC1* mutants have fewer branches in the inflorescence (Li et al., 2003), and the *LAX2 MOC1* double mutant lacks branches entirely (Tabuchi et al., 2011). Mutants in *MOC1* orthologs have not been described in other grasses.

Likewise, BARREN STALK FASTIGIATE1 (BAF1) in maize, an AT-hook domain DNA binding protein with a plant-specific Plant and Prokaryote Conserved domain, positively regulates *BA1* although the two do not interact directly (Gallavotti et al., 2011). In *BAF1* mutants, axillary meristems are fused to the axis, but bracts are unaffected (Gallavotti et al., 2011). BAF1 may also regulate axillary meristems directly, bypassing *BA1* (Matthes et al., 2019). The BAF1 ortholog in rice affects floral development and is known as *PALEALESS1* or *DEPRESSED PALEA1* (*DP1*; Jin et al., 2011). However, a branching phenotype is not reported in rice, nor is a floral phenotype in maize.

Expression of *RAMOSA2* (*RA2*, orthologous to barley *HvRA2*(*VRS4*) and rice *OsRA2*) marks the position of primary and secondary BMs in maize, sorghum, rice, and barley (Bortiri et al., 2006; Figure 4). RA2 is a Lateral Organ Boundaries (LOBs)-domain transcription factor that is conserved in grasses (Bortiri et al., 2006; Koppolu et al., 2013), and has a grass-specific sequence upstream of the LOB domain (Koppolu et al., 2013). In normal development, the IM in both maize and barley produces short lateral branches each of which produces only two (maize) or three (barley) spikelets (Figure 3); in the maize and barley literature, the meristems producing these short branches are known as a spikelet pair meristem and a triple mound, respectively. Mutations in *RA2* and *HvRA2* permit the short branches to continue growth, leading to a branch with unpaired spikelets in maize (Bortiri et al., 2006), and a branch-like central spikelet and fertile lateral spikelets in barley (Koppolu et al., 2013). This continued growth reflects a delay in terminal spikelet formation, also described as loss of determinacy. In contrast, downregulation of *OsRA2* did not affect branching but pedicel length increased, indicating that the normal function of the protein in rice is to prevent growth of specific tissues, but possibly not inflorescence branches (Lu et al., 2017). Branch length was not affected, although overexpression of *OsRA2* reduced the number of secondary branches.

## Opposing regulatory gradients of miR156-SPL control branching

Some developmental decisions can be described as transitions and gradients, with the gradients often running in opposition to each other. microRNAs and their targets have become well known for setting up such opposing gradients. For example, the microRNA *miR156* is upregulated by SPL proteins; it then cleaves the corresponding SPL transcript in a negative feedback loop, a process initially elucidated in vegetative to reproductive phase change in Arabidopsis and maize (Chuck et al., 2007a, 2007b; Poethig, 2009; Wu et al., 2009). In Arabidopsis, as *miR156* expression decreases, *SPL3* expression goes up, increasing expression of *LFY*, *AP1*, and *FUL* (Yamaguchi et al., 2009).

In grasses, several SPL proteins and their regulatory microRNAs control the transition from branching to spikelet production. The rice genome includes 19 *OsSPL* loci, 11 of which could be targets of *miR156* based on sequence comparisons (Xie et al., 2006; Yang et al., 2008); comparable numbers in maize are 30 and 18, respectively. Many rice SPL loci were discovered initially as quantitative trait loci in studies aiming to improve grain number; because of that history, many have been named more than once in the literature. A full list of alternative gene names is in Supplemental Table S1. Among the loci with *miR156* binding sequences are *OsSPL6* (*ZmSBP6, 17*), *OsSPL8* (*ZmLG1*), *OsSPL13* (*ZmSBP13*, *29*), and *OsSPL16*, *OsSPL18* (the latter two co-orthologous to TEOSINTE GLUME ARCHITECTURE1 (*TGA1*), NEIGHBOR OF TGA1 (NOT1), and *ZmSBP5*; Wei et al., 2018). Other highly expressed OsSPL loci include *OsSPL7*, *OsSPL14*, and *OsSPL17* (Wang et al., 2015).

**Figure 5:**
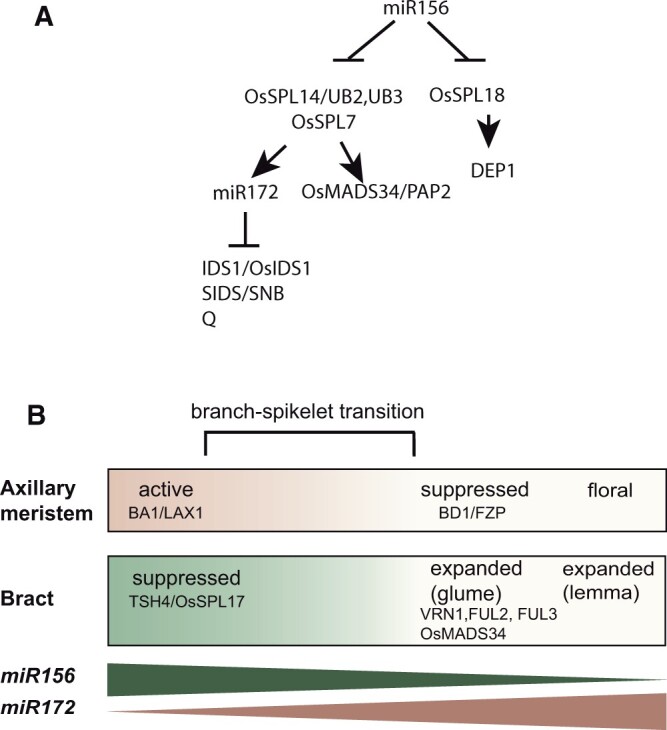
A, *miR156-SPL-miR172-AP2-like* regulatory networks. B, Developmental window showing the transition from axillary BMs to spikelet formation, with major genes marking each stage and opposing gradients of microRNAs. Species- and clade-specific inflorescence morphology is influenced by the developmental timing of the transition; shorter time causes a faster transition to glume production which in turn leads to fewer branches and vice versa. In rice, transition time appears to vary continuously across the inflorescence; in maize, transition time is bimodal (long and short, but nothing in between); in barley, transition time is unimodal, only short. Portions of the figure are redrawn from Wang et al., (2015, Supplemental Figure S16).

*OsSPL14* (orthologous to maize *UB2* and *UB3*) has received particular attention (Jiao et al., 2010; Miura et al., 2010). Increased transcription of *OsSPL14* leads to more primary inflorescence branches (Huang et al., 2016), and heterozygotes were strongly over-dominant for yield (reflecting higher ultimate numbers of spikelets; Figure 5A). Mutation of the *miR156* binding site also increased *OsSPL14* expression and yield (Jiao et al., 2010). Overexpression of *OsSPL14* or inhibition of *miR156* both led to early transition from BMs to SMs. Consistent with this interpretation, expression of *FRIZZY PANICLE1* (*FZP1*; a spikelet marker, see below) was higher and *FZP* was expressed in meristems that might otherwise have produced branches (Wang et al., 2015). Mutant phenotypes of *OsSPL14* and *OsSPL17* are similar, with double mutants (RNAi) showing enhanced effects.

The complex of APO1, an F-box protein and APO2 (RFL), a homolog of Arabidopsis LEAFY, also delays the transition from BMs to producing glumes (Ikeda et al., 2005; Ikeda-Kawakatsu et al., 2009, 2012). When the LFY homologs ZFL1 and ZFL2 are mutated in maize, the transition to normal tassel branches is also delayed and axillary meristems develop in husk leaves of the ear (Bomblies et al., 2003).

The direct interaction of APO1 and APO2 appears to be conserved in plants, having been demonstrated in Arabidopsis (orthologs UNUSUAL FLORAL ORGANS and LFY, respectively), rice, and barley (Chae et al., 2008; Kyozuka, 2014; Selva et al., 2021). However, the two proteins delay the transition to spikelet formation in rice and maize in whereas they promote flower formation in Arabidopsis (Kyozuka, 2014). Their role in barley is still different, in that disruption of HvLFY does not affect inflorescence architecture nor does it affect expression of *APETALA1*/*FRUITFULL* (*FUL*)-like (*FUL*-like) genes, as might be expected if it affected spikelet formation (Selva et al., 2021). WFL (ortholog of LFY in wheat) is expressed in the bracts (lower ridge) below the spikelets, but not in the spikelets themselves (Shitsukawa et al., 2006); its mutant phenotype is unknown.

## Control of secondary and higher order branches is species- and position specific

Both the number and morphology of secondary and higher order branches vary among cereal grasses (Figure 3), among genera within a tribe (e.g. Cynodonteae; Pilatti et al., 2019), and among species within a genus (e.g. *Setaria*; Doust and Kellogg, 2002). The range of natural variation suggests that genetic control of secondary branches may be partially independent of the control of primary branches, a hypothesis supported by genetic data.

### Secondary branching in rice

Primary and secondary branches are controlled independently in rice, even though all branches are morphologically similar (Harrop et al., 2019; Bai et al., 2021). For example, double mutants of *LAX1LAX2* (described above), have no visible defect in primary branches but lack secondary branches and spikelets altogether (Tabuchi et al., 2011). Allelic variation has been explored extensively in *FZP1* (see also below), an AP2/EREBP transcription factor (Bai et al., 2017; Fujishiro et al., 2018; Huang et al., 2018; Wang et al., 2020), where mutations in the promoter affect the binding of transcription factors and thereby expression levels. An allele of *FZP1* originally known as *CONTROL OF SECONDARY BRANCH1* acts particularly on secondary branches (Huang et al., 2018). A small deletion in the promoter of *FZP1* reduces binding of the ARF OsARF6 and reduces *FZP* expression, leading to increased cell division and more secondary branches, but no changes in primaries.

In addition, FZP1 interacts with and is degraded by NARROW LEAF 1 (NAL1), a serine/cysteine protease. Downregulation of *FZP1* along with upregulation of *NAL1* improved yield in rice (Huang et al., 2018), again by increasing secondary branches. *NAL1* is expressed throughout the plant, particularly in vascular tissues (Qi et al., 2008), whereas *FZP1* is expressed only in the inflorescence.

Knockout of *OsSPL18* significantly reduces the number of secondary branches, and *OsSPL18* is itself cleaved by *OsmiR156k* (Yuan et al., 2019). OsSPL18 binds to the promoter of *DENSE and ERECT PANICLE1* (a G-protein γ subunit; Xing and Zhang, 2010; Liu et al., 2021) and activates it (Yuan et al., 2019), thereby increasing cell numbers. Mutations in *OsSPL9*, the gene underlying the mutant *LESS GRAIN NUMBER5*, exhibited less than half the number of secondary branches as wild-type *indica* lines, although primary branch number was unaffected (Hu et al., 2021).

Overexpression of *RICE CENTRORADIALIS1* and *2* (*RCN1*, *RCN2*), homologs of *CENTRORADIALIS*/*TERMINAL FLOWER1*, led to increased panicle branching in rice (Nakagawa et al., 2002; Wang et al., 2015), indicating that the primary function of the RCNs is to reduce branching, perhaps by accelerating the transition to spikelet formation. Overexpression of *RCN* rescued the effects of *OsSPL14* and *OsSPL17* RNAi lines on secondary branches but did not affect primary branches (Wang et al., 2015).

OsMADS34/PANICLE PHYTOMER2 (PAP2) in rice also controls the relative numbers of primary and secondary branches (Gao et al., 2010; Kobayashi et al., 2010) with the normal function to reduce numbers of primary branches. The effect on secondary branching is unclear, with some mutations leading to more secondary branches and hence spikelets (Kobayashi et al., 2010), while others reported mutations lead to fewer (Gao et al., 2010).

The regulatory networks controlling secondary branch formation in rice may be relevant in other species with open branching inflorescences such as the closely related genus *Zizania* (North American wild rice) or the distantly related genera *Panicum* (switchgrass) or *Megathyrsus* (guinea grass). However, other species have distinct architecture and are hard to compare to rice (Figure 3).

### Secondary branching in maize: spikelet pairs

Maize produces long and short inflorescence branches. In the tassel, the first-formed primary branches are long, whereas later ones are short, producing exactly two spikelets (spikelet pairs; Figure 3). In these, one spikelet is lateral (i.e. a secondary branch) and the other is terminal. Primary branches in the ear are also short (spikelet pairs) as are secondary branches in the tassel.

The maize branch regulator RA2 is genetically upstream of *RA1*, which has a similar mutant phenotype in which spikelet pairs are converted to longer branches, often with single spikelets (Vollbrecht et al., 2005; Bortiri et al., 2006; Figure 4). RA1 is a C2H2 zinc-finger transcription factor containing two Ethylene-responsive element binding factor-associated Amphiphilic Repression (EAR) domains (Vollbrecht et al., 2005). RA1 interacts directly with RAMOSA ENHANCER LOCUS1 (REL1), orthologous to ABERRANT SPIKELET AND PANICLE1 (ASP1) in rice (Gallavotti et al., 2010; Tanaka et al., 2013). REL1/ASP1 is a protein with an AT-hook domain similar to TOPLESS in Arabidopsis and is thought to be a transcriptional co-repressor. However, because rice lacks a *RA1* locus (Vollbrecht et al., 2005), the interactors of ASP1 are unclear.

RA3, a trehalose-6-phosphate phosphatase (TPP) in maize, is genetically independent of RA2 although it also affects the short branch/spikelet pair meristem (Satoh-Nagasawa et al., 2006; Figure 4). TPP may link sugar metabolism to signaling, since other TPPs repress *SUCROSE-NON-FERMENTING1-RELATED KINASE1* and also *miR156* which in turn negatively regulates SPL proteins (Eveland and Jackson, 2012; Tsai and Gazzarrini, 2014). However, RA3 co-localizes with RNA POLYMERASE II in nuclear speckles (Demesa-Arevalo et al., 2021), and an *RA3* construct lacking phosphatase activity will still complement the *ra3* mutant (Claeys et al., 2019). Together these observations suggest a transcriptional regulatory role for RA3 separate from its role as an enzyme.

### Secondary branching in barley: triplets of spikelets

*RA1* and *RA3* are absent from genomes in the BOP clade, so rice, barley, wheat, and other related species must have distinct pathways regulating higher order branching (Vollbrecht et al., 2005; Doust, 2007; Kellogg, 2007). In barley, the primary branches are short and terminate in a spikelet, but before terminating they produce exactly two lateral (secondary branch) spikelets. While this complex of three spikelets is formed from a triple meristem, in barley relatives such as *Elymus* and *Leymus* the number of secondary spikelets varies from one to three depending on the species (POWO, 2021).

The controls of secondary (lateral) branching in barley reflect a complex network involving branching, glume formation, and floral organ development (Gauley and Boden, 2019). Branching itself (i.e. formation of the laterals) is governed by HvRA2 but proteins regulated by HvRA2 differ from those of maize RA2, which is unsurprising given the lack of RA1 and RA3 in barley (Figure 4). As in maize, a TPP protein is genetically downstream of HvRA2, but the barley TPP protein is HvSRA, which is not orthologous to RA3 but rather belongs to the SISTER OF RA3 (SRA) clade of TPPs that is conserved in grasses. SRA is apparently not involved in inflorescence development in maize (Satoh-Nagasawa et al., 2006). HvRA2 upregulates *VRS1/HOX1*, a homeodomain leucine zipper transcription factor that is the result of a gene duplication specific to Triticeae (Komatsuda et al., 2007; Koppolu et al., 2013; Sakuma et al., 2019). The HOX1/HOX2 clade is in turn sister to orthologs of GRASSY TILLERS1 in maize (Whipple et al., 2011). Recent work on barley MADS-box transcription factors also suggests they regulate inflorescence branching, possibly in response to temperature, in addition to their expected function in floral organ identity (Kuijer et al., 2021; Li et al., 2021a, 2021b, 2021c). Thus, despite the conserved LOB-domain transcription factors (RA2 and HvRA2) and the involvement of a TPP protein, the controls of spikelet pairs and lateral branches in Triticeae differ from those in other grasses.

## Spikelet bracts (glumes) expand, axillary bud growth is suppressed

### Glume production is common but not a necessary marker of a transition to floret production

The shift from a BM with suppressed bracts and active axillary meristems to a spikelet-producing meristem is clear in many grasses, with the SM producing exactly two macroscopic bracts (glumes) with suppressed axillary meristems, followed by one or more large bracts (lemmas) subtending FMs. However, that transition may be protracted, with some species producing more than two glume-like structures. For example, the glumes in rice are tiny and known as rudimentary glumes (Figure 2). Distal to the glumes are two structures in the position of florets that also fail to produce axillary FMs. Although many lines of evidence support the inference that these are sterile lemmas, they are expanded bracts without an axillary meristem so share some characteristics with glumes. Mutations in *G1*/*LONG SLENDER LEMMA1* (Yoshida et al., 2009; Yang et al., 2020) shift the size and cellular morphology of the sterile lemmas to look more like true lemmas, whereas mutations in other genes lead to stronger similarity between the sterile lemmas and rudimentary (true) glumes (summarized by Ren et al., 2018; Xu et al., 2020). However, none of the mutants leads to production of an axillary FM, as would be expected if the sterile lemmas were fully converted to true lemmas. Other species (e.g. *Chasmanthium*) have multiple sterile lemmas, whereas many Bambusoideae bear pseudo-spikelets, which are subtended by glume-like structures with axillary meristems that themselves produce spikelets, somewhat reminiscent of *FZP* mutants (see below).

Conversely, the absence of glumes is well documented in some species of grasses, including many members of Oryzeae (some of which also lack sterile lemmas), as well as *Nardus* and *Lygeum* in the tribe Nardeae (subfamily Pooideae), tribe Orcuttieae (Chloridoideae), *Piresia* (Bambusoideae), and others (Kellogg, 2015).

The relative timing of glume production determines the overall architecture of the inflorescence and thereby the potential for seed production. In all grasses studied, meristems in the axils of glumes are suppressed by BRANCHED SILKLESS1 (BD1; maize)/FZP1; rice), orthologs of which have been characterized in *B.* *distachyon*, barley, and wheat (Chuck et al., 2002; Komatsu et al., 2003; Zhu et al., 2003; Derbyshire and Byrne, 2013; Dobrovolskaya et al., 2015; Poursarebani et al., 2015). Meristems form in the axils of glumes in *BD1/FZP1* mutants, and these axillary meristems each produce glumes with axillary meristems. FZP is thus central to the transition from BM with suppressed bracts to SM with suppressed axillary meristems. The broad phylogenetic distribution of these systems indicates that the function of FZP1 orthologs is likely conserved among all spikelet-bearing grasses.

BD1/FZP1 is a transcription factor with a single AP2 domain, and part of the AP2/ERF clade similar to PUCHI in Arabidopsis (Chandler, 2018), which also specifies axillary meristems in the inflorescence (Karim et al., 2009). Unlike BD1/FZP1, PUCHI appears to promote axillary meristem (flower) growth rather than suppress it. The sequence of the BD1/FZP AP2 domain is conserved across grasses. Two FZP1 homologs have been identified in the genome of *Pharus latifolius*, a member of the subfamily Pharoideae that is sister to all other spikelet-bearing grasses (Ma et al., 2021; Figure 1), although only one gene is expressed in young inflorescences.

### Transition from producing suppressed bracts to producing glumes controls numbers of branches, particularly secondaries

Within the inflorescence and along each branch, gradients of gene regulation, particularly via *miR172* and *euAP2*-like genes, control the transition from BM production (suppressed bracts) to SM production (expanded bracts; Wang et al., 2015; Figure 5B). The relative speed of this transition controls the architecture of the inflorescence (Kyozuka, 2014).

*euAP2-*like genes have two AP2 domains (Kim et al., 2006), rather than the single domain found in *FZP*. In addition, nearly all genes in this group also have *miR172* binding sites (Seetharam et al., 2021) and *miR172* expression opposes expression of *AP2-*like genes (Figure 5A). The *AP2-*like*-miR172* interaction has been investigated for its role in specifying the number of florets per spikelet (e.g. Chuck et al., 2007, 2007b, 2008; Zhu et al., 2009), but another important role of AP2-like-*miR172* is to delay production of glumes, thereby prolonging branching.

In single and double mutants of the rice *AP2*-like genes *INDETERMINATE SPIKELET1* (*OsIDS1*) and *SUPERNUMERARY BRACT* (*SNB*), the IM and BMs were converted precociously to spikelets, leading to fewer branches (both primary and secondary), with the number varying in a dose-dependent manner (Lee and An, 2012). Mutant spikelets had extra rudimentary glumes, indicating that the meristem had made a transition from producing suppressed bracts (as in a branch) to producing glumes (as in a spikelet), but had failed in the subsequent transition to FM production (Lee and An, 2012). Consistent with this interpretation, *FZP* expression appeared earlier in BMs of the mutants than in wild-type. Overexpression of *miR172* in rice produced a phenotype similar to that of the *OsIDS1 SNB* double mutant. Mutations in the orthologous genes in maize (*IDS1* and *SISTER OF IDS* [*SIDS*]) showed similar phenotypes, with fewer branches and extra glumes (Chuck et al., 2008).

In wheat, the *IDS* ortholog is the domestication gene *Q* (Seetharam et al., 2021), which is also regulated by *miR172* (Debernardi et al., 2017). The transition to forming glumes is particularly obvious in wheat because the glumes have prominent keels, shorter awns, and more sclerenchyma than lemmas. Reduction of *miR172* led to higher levels of *Q* (*AP2-5*) and greater similarity between glumes and lemmas. Conversely high levels of *miR172* and loss-of-function *AP2-5* led to sterile lemmas. In the lowermost spikelets, the transition between glumes and lemmas appeared particularly malleable, such that more *miR172* and less *AP2-5* could lead to glume-like organs in the position of lemmas (i.e. sterile lemmas).

Some SPL proteins, such as OsSPL7 and OsSPL14, directly regulate *miR172* in rice and accelerate the transition to producing glumes (Wang et al., 2015; Figure 5A). The mutant phenotype caused by overexpression of either *SPL* locus was returned to normal by knockdown of *miR172*. Overexpression of *RCN1* and *RCN2* also led to increased panicle branching in rice by delaying the transition to SMs (Nakagawa et al., 2002; Wang et al., 2015).

TAWAWA1 is an ALOG protein that controls the timing of IM degeneration in rice and also the transition from BM to spikelet formation (Yoshida et al., 2013). Kyozuka (2014) has proposed that TAW1 is central to meristem maintenance in the IM and BMs, with lower levels leading to early IM abortion and accelerated transition from BMs to spikelet formation. TAW1 regulates *SHORT VEGETATIVE PHASE* (*SVP*) genes, which encode MADS-box transcription factors (Arora et al., 2007; Lee et al., 2007).

## SM identity reconsidered

The existence of axillary signaling centers and gradients of developmental signals suggests that SM identity may be achieved by the confluence of several gene expression patterns that, when overlapping, produce the stereotypical grass spikelet. However, such patterns could also activate SM identity genes that are both necessary and sufficient to specify a structure as a spikelet. SEP-like and FUL-like MADS-box genes are good candidates for SM identity controls (Bommert and Whipple, 2018) as are the SPL proteins TGA1 and NOT1 (Preston et al., 2012).

MIKC-type MADS-box genes are well known as homeotic selector genes and some aspects of their function, particularly B-class (generally inner perianth and stamen expression patterns) and C-class (generally stamen and carpel expression) are conserved between dicots and grasses (Bommert et al., 2005). In contrast, the A-class function, originally thought to specify sepal identity and attributed to AP1, has been elusive (Litt and Irish, 2003; Litt, 2007). Grasses lack an ortholog of the dicot AP1 and instead have three loci that are more closely related to FUL in dicots (Preston and Kellogg, 2006, 2007). The three proteins, VERNALIZATION1 (VRN1; unrelated to the Arabidopsis protein of the same name), FUL2, and FUL3, affect plant height and flowering time in wheat, rice, Brachypodium, and Setaria (Yan et al., 2003; Kobayashi et al., 2012; Ream et al., 2014; Li et al., 2016; Woods et al., 2016; Li et al., 2019a, 2019b; Yang et al., 2021).

*VRN1* and *FUL2* are expressed throughout the spikelet (glumes plus florets) in *Lolium* (ryegrass), *Triticum*, *Hordeum*, *Avena*, and *Setaria* (Gocal et al., 2001; Preston and Kellogg, 2008; Preston et al., 2009; Alonso-Peral et al., 2011; Yang et al., 2021), as well as being expressed in the IM and BM. Knockout of *VRN1FUL2* or *VRN1FUL2FUL3* in both genomes of tetraploid wheat specifically affected SM identity, consistent with their expression patterns (Li et al., 2019a, 2019b). In the mutants, the lower ridge expanded to form a leaf and the spikelet (the sole product of a primary branch) was replaced by a leafy tiller-like structure. Thus the ability of the IM to position and form the subtending bract is not compromised but spikelet identity is disrupted. Having either VRN1 or FUL2 is enough to make a terminal spikelet and repress the wheat homologs of *RCN*; FUL3 controls timing of (accelerates) terminal flower production (Li et al., 2019a, 2019b). The balance between the FUL-like proteins and SVP proteins determines whether spikelets form normally in wheat, or whether they develop into tiller-like branches (Li et al., 2021a, 2021b, 2021c).

The rice proteins OsMADS5 and OsMADS34 (=PANICLE PHYTOMER2) add another layer of regulation. Like wheat *VRN1 FUL2 FUL3* null triple mutants, the rice *OsMADS14 OsMADS15 OsMADS18 PAP2* quadruple mutant replaces primary branches with vegetative tiller-like structures (Kobayashi et al., 2012; Li et al., 2019a, 2019b). In rice, OsMADS5 and OsMADS34 directly regulate *RCN4* and accelerate the transition to spikelet production. Double mutants of *OsMADS5 OsMADS34* or *OsMADS34 RCN4* produce more branches, including secondary, tertiary, and even quaternary branches (Zhu et al., 2021a, 2021b). *OsMADS34* promoters also contain SPL binding motifs, and *OsMADS34* is directly regulated by OsSPL14 (Wang et al., 2015).

Mutations in *OsMADS34* have no effect on rudimentary glumes, although the gene is expressed there; sterile lemmas in the mutants are morphologically similar to true lemmas but still do not produce axillary FMs (Gao et al., 2010). LACKING RUDIMENTARY GLUME 1 (LRG1) is also involved in glume and sterile lemma identity. In an unexpected example of regulatory convergence, LRG1 is a C2H2 transcription factor similar (although not orthologous) to RA1, with similar EAR repression domains and interactions with a TOPLESS-like protein (Xu et al., 2020). A full discussion of rice spikelet morphology is beyond the scope of this paper but will be interesting to pursue in the future.

Lee and An (2015) noted that expression of *FUL-*like MADS-box genes was unaffected in *SNB OsIDS* double mutants. One interpretation is that SNB and OsIDS are needed to establish the domain within which the FUL-like proteins can specify spikelet identity. Such an interpretation awaits additional data.

The SPL protein TGA1 acquired its current expression domain in the spikelet-bearing grasses (Preston et al., 2012) and is another candidate for conferring spikelet identity. In all grasses examined, it is expressed in the florets and both glumes. However, it is expressed only in the flower (not the floral bracts) of the grass outgroup *Joinvillea ascendens*. Thus, the grass expression pattern represents an expansion of floral control to encompass the bracts. Regulation of *TGA* expression has not been explored, although the *miR156* binding site is conserved among grasses and their outgroups (Preston et al., 2012).

## Summary: conservation and diversity

The controls of inflorescence architecture are strikingly similar among many grasses (Figure 5B). Auxin transport and signaling use orthologous proteins retaining similar biochemical functions, interactions, and developmental roles in most species. Likewise, conserved mechanisms specify the position and development of suppressed bracts via SBP proteins such as OsSPL18 and TSH1. BA1 and BA2 and their regulators and targets also appear conserved in positioning and delimiting axillary meristems. Spikelets are marked by formation of glumes; suppression of their axillary meristems is controlled by FZP/BD. Timing of transitions from IM to BM to SM is controlled by opposing gradients of *miR156-SPL-miR172-AP2-*like gene expression. This unifying mechanistic picture offers insights that may be applicable to less well-studied crops, as well as wild grasses.

Despite this broad similarity, many other mutant phenotypes have been observed only in a single species; it is unclear whether such gene functions are indeed phylogenetically restricted or if data on other species are simply lacking. For example, DP1 is the rice homolog of BAF1 and may have a different developmental role; however, the requisite data are not available. Allelic variation in *FZP* has been dissected carefully in studies in rice attempting to maximize yield, but no comparable data are available for *BD1* in maize. Likewise, sets of genes control secondary branches (products of the primary BM) independent of primary branches (products of the IM) in rice, indicating that these two meristems are developmentally distinct, but few comparisons are available for other species.

In other cases, whole-genome sequences show that critical proteins that are critical for one species are simply absent in others. For example, *RA1* and *RA3* are not present in genomes of species of the BOP clade, implying that their function in rapid transition to a terminal spikelet in the short-branch (spikelet pair) meristems of maize and sorghum may be species- or clade specific. Genes that are genetically downstream of HvRA2 also differ from those genetically downstream of RA2 in maize, suggesting that each gene network may be only applicable in close relatives of barley or maize, respectively. Such presence–absence variation is only beginning to be explored.

In the future, we can anticipate identifying additional regulatory networks that make grass inflorescences so similar as well as the network components that make individual species morphologically distinct. The conserved components may be expected in all grasses, including orphan crops, whereas the variable components await analysis in disparate species.

## Supplemental data

The following material is available in the online version of this article.

**Supplemental Table S1.** Protein-coding genes discussed in this article.

## Supplementary Material

koac080_Supplementary_Table_S1

## Notes

The author responsible for distribution of materials integral to the findings presented in this article in accordance with the policy described in the Instructions for Authors (https://academic.oup.com/plcell) is: Elizabeth A. Kellogg (ekellogg@danforthcenter.org).
